# Improved Photoresponse Characteristics of a ZnO-Based UV Photodetector by the Formation of an Amorphous SnO_2_ Shell Layer

**DOI:** 10.3390/s21186124

**Published:** 2021-09-13

**Authors:** Junhyuk Yoo, Uijin Jung, Bomseumin Jung, Wenhu Shen, Jinsub Park

**Affiliations:** 1 Department of Electronics & Computer Engineering, Hanyang University, Seoul 04763, Korea; loveyoo16@naver.com; 2 Department of Electronic Engineering, Hanyang University, Seoul 04763, Korea; qwpoiu1002@hanyang.ac.kr (U.J.); bomseumin@hanyang.ac.kr (B.J.); moonho@hanyang.ac.kr (W.S.)

**Keywords:** core–shell structure, surface passivation, nanostructure, UV photodetector

## Abstract

Although ZnO nanostructure-based photodetectors feature a well-established system, they still present difficulties when being used in practical situations due to their slow response time. In this study, we report on how forming an amorphous SnO_2_ (a-SnO_2_) shell layer on ZnO nanorods (NRs) enhances the photoresponse speed of a ZnO-based UV photodetector (UV PD). Our suggested UV PD, consisting of a ZnO/a-SnO_2_ NRs core–shell structure, shows a rise time that is 26 times faster than a UV PD with bare ZnO NRs under 365 nm UV irradiation. In addition, the light responsivity of the ZnO/SnO_2_ NRs PD simultaneously increases by 3.1 times, which can be attributed to the passivation effects of the coated a-SnO_2_ shell layer. With a wide bandgap (~4.5 eV), the a-SnO_2_ shell layer can successfully suppress the oxygen-mediated process on the ZnO NRs surface, improving the photoresponse properties. Therefore, with a fast photoresponse speed and a low fabrication temperature, our as-synthesized, a-SnO_2_-coated ZnO core–shell structure qualifies as a candidate for ZnO-based PDs.

## 1. Introduction

Ultraviolet (UV) photodetectors (PDs) have received much attention due to their diverse applications, such as in flame detection, optical communication, biological analysis, and emitter calibration [1,2,3]. Several metal oxide (MO) materials have been recommended for use in UV PDs, such as ZnO [4,5], TiO_2_ [6], and SnO_2_ [7,8]. Among the various suggested MO materials, low-dimensional ZnO has been considered a key material for UV PDs due to its large surface–volume ratio and Debye length [9,10]. However, until now, ZnO-based UV PDs have typically exhibited slow light response speeds, which can be a critical drawback for the application of high-performance PDs [11,12,13,14]. As summarized in Table S1, the rise time and the fall time of most ZnO-based PDs are of the order of several seconds. Although some ZnO-based PDs have shown response times of milliseconds or microseconds, the fabrication processes used to improve the response properties require a high temperature of above 800 °C or a high-vacuum environment [15,16,17,18]. The slow light response speeds and high photoconductive gain exhibited by ZnO-based UV PDs are strongly related to the oxygen-mediated detection mechanism [19,20,21]. Accordingly, there is a trade-off between the high photoconductive gain and the fast light response speed. To improve their rise and fall times without degrading the photoconductive gain, introducing a shell layer to the ZnO core, such as AIN [18], Al_2_O_3_ [22], InN [23], SnO_2_ [24], PVA [25], CuCrO_2_ [26], or TiO_2_ [27], may be a promising solution, resulting in an improvement in the overall performance of PDs. However, forming the shell layer of these materials requires a high temperature and is a complex and expensive process. Therefore, it is necessary to study the facile process for the formation of the shell material. SnO_2_ can be used as a shell material for ZnO due to its excellent properties, including a wide direct band gap, high transparency with high mobility, and excellent stability [28,29].

Here, we demonstrate a UV PD with a ZnO/a-SnO_2_ core–shell nanorods (NRs) structure that shows improved photoresponse speeds. The ZnO/a-SnO_2_ core–shell nanorods’ (NRs) structure was synthesized using a cost-effective, solution-based method at a relatively low temperature (below 200 °C). The effects on the UV PD’s performance of forming an a-SnO_2_ layer on the ZnO NRs were investigated by characterizing fabricated ZnO/a-SnO_2_ UV PDs. Introducing the a-SnO_2_ layer was an effective way to improve the performance of ZnO UV PDs. The possible mechanism for the effect of the a-SnO_2_ shell as a surface passivation layer is also discussed.

## 2. Materials and Methods

### 2.1. Materials

All the chemicals were used without further purification. Zinc acetate dehydrate (Zn(CH_3_CO_2_)_2_∙2H_2_O, >99.0%), zinc nitrate hexahydrate (Zn(NO_3_)_2_∙6H_2_O, >99.0%), ethanolamine (C_2_H_7_NO, >99.0%), 2-methoxyethanol (C_3_H_8_O_2_, >99.3%), and potassium stannate trihydrate (K_2_SnO_3_∙3H_2_O, 99.9%) were purchased from Sigma-Aldrich (St. Louis, MO, USA). Hexamethylenetetramine (HMTA, (CH_2_)_6_N_4_, 99.0%) and urea (CO(NH_2_)_2_, 99.0%) were purchased from Samchun Chemicals (Seoul, Korea) and hydrochloric acid (HCl, 35%) was purchased from Daejung Chemicals & Metals (Siheung, Korea).

### 2.2. Fabrication of ZnO/a-SnO_2_ Core–Shell Nanorods-Based Photodetectors

**Figure 1:**
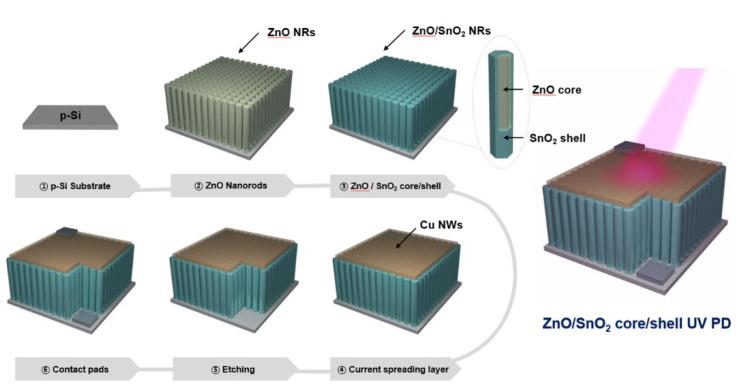
The fabrication process of the ZnO/SnO_2_ core–shell photodetector.

Figure 1 shows the overall fabrication process of the UV PDs with a ZnO/a-SnO_2_ core–shell NRs structure. At first, the ZnO seed solution was prepared by adding 0.025 M Zn(NO_3_)_2_∙6H_2_O and 0.025 M ethanolamine to 20 mL of C_3_H_8_O_2_ and then stirring for 1 h. After spin-coating a few drops of the ZnO seed solution on a p-type silicon (p-Si) substrate, an annealing process was conducted at 300 °C for 10 min to form the ZnO seed layer. In turn, the ZnO growth solution was prepared by dissolving 0.05 M Zn(NO_3_)_2_∙6H_2_O and 0.05 M HMTA in 100 ml of deionized (DI) water and then stirring for 10 min. Subsequently, the ZnO seed-coated p-Si was immersed into the growth solution and hydrothermal growth was carried out at 95 °C for 3 h. The sample was then washed with deionized (DI) water and the ZnO NRs were successfully grown on the p-Si substrate. In addition, to form the SnO_2_ shell layer on the ZnO NRs, the growth solution of the SnO_2_ was prepared by mixing 0.01 M K_2_SnO_3_∙3H_2_O with 0.1 M CO(NH_2_)_2_ in 20 mL of a solvent that consisted of a mixture of ethanol and DI water (60 vol% ethanol) and stirring for 20 min. The SnO_2_ growth solution was poured into the 30 ml Teflon-lined stainless-steel autoclave and the as-synthesized ZnO NRs/p-Si was immersed in the solution. Hydrothermal growth was then allowed to proceed at 165 °C for 1 h. After cooling to room temperature and washing with DI water, the ZnO/SnO_2_ core–shell NRs were successfully obtained. To fabricate the UV PD, copper nanowires (Cu NWs) were prepared by following the synthesis sequence described in the literature [30]. The prepared Cu NWs were transferred onto the as-synthesized ZnO/SnO_2_ NRs by a vacuum filtration process, which served as the carrier transport layer [31]. An etching process for the contact pads area was carried out using the HCl solution, and the Ag contact pads were sequentially formed. Finally, the ZnO/SnO_2_ core-shell NRs UV PD was successfully fabricated.

### 2.3. Characterization

The morphology and chemical composition of the as-synthesized NRs were investigated using a scanning electron microscope (SEM) (JSM-6700F, JEOL, Tokyo, Japan) and a field emission transmission electron microscope (FE-TEM) (JEM-F200 (TFEG), JEOL, Tokyo, Japan). The crystal structure of the as-synthesized NRs was obtained using an X-ray diffractometer (XRD) (New D8 Advance, Bruker, Billerica, MA, USA). To analyze the optical properties of the as-synthesized NRs, UV–Vis–NIR (Cary 5000) spectroscopy and photoluminescence (PL) (MonoRa750i, Dongwoo Optron) measurements were performed. I–V characteristics and time-domain photoresponse (I–t) were measured under 365 nm (610 μW/cm^2^) and 254 nm (400 μW/cm^2^) UV light sources using a Keithley 2400 source meter unit at the customized probe station in the dark shield box (MSD2, MS tech.). I–V characteristics were measured in the range of −5 V to 5 V, and the I-t characteristics were measured at a fixed bias condition of −5 V. All electrical measurements were executed at room temperature and air atmosphere.

## 3. Results and Discussion

**Figure 2:**
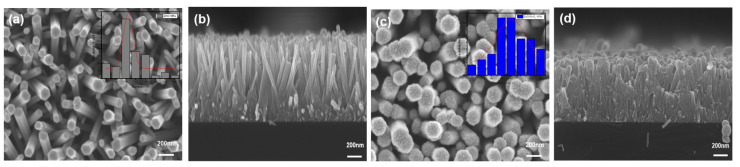
SEM images of the ZnO PD and ZnO/SnO_2_ PD. (**a**) SEM image of ZnO NRs. The inset shows the diameter size distribution of ZnO NRs. (**b**) Cross-sectional view of ZnO NRs. (**c**) SEM image of ZnO/SnO_2_ NRs. The inset shows the diameter size distribution of ZnO/SnO_2_ NRs. (**d**) Cross-sectional view of ZnO/SnO_2_ NRs.

The morphologies of as-synthesized ZnO NRs and ZnO/SnO_2_ core–shell NRs were observed using an SEM, as shown in Figure 2. Figure 2a,b shows the top and cross-sectional views, respectively, of the SEM images obtained from the as-synthesized ZnO NRs, which clearly show that the hexagonally structured ZnO NRs with a vertical alignment were successfully synthesized on the Si substrate by the solution process. The mean diameter and length of the synthesized ZnO NRs were about 100 ± 2.8 nm and 1 μm, respectively. After the synthesis of the ZnO NRs, the SnO_2_ shell layer was subsequently formed on the core ZnO surface, as shown in Figure 2c (top view) and Figure 2d (cross-sectional view). The measured mean diameter of the ZnO/SnO_2_ NRs was approximately 180 ± 3 nm. From the morphological change and the increase in diameter from the bare ZnO NRs to the ZnO/SnO_2_ NRs, we can speculate that the ZnO/SnO_2_ core–shell structure was successfully formed, and the thickness of the SnO_2_ shell layer was approximately 40 nm.

**Figure 3:**
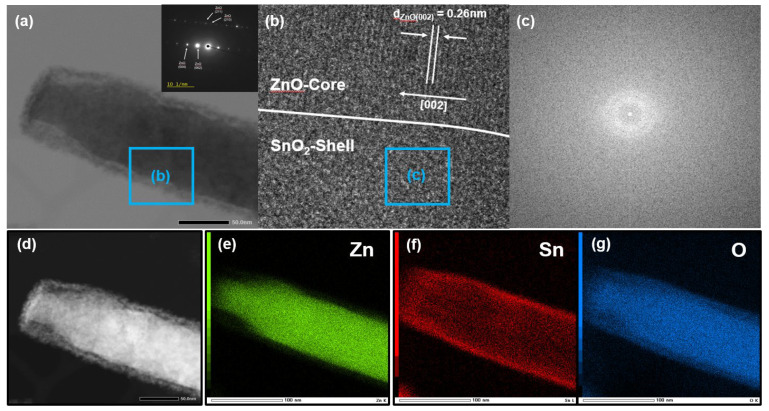
TEM images of ZnO/SnO_2_ NR. (**a**,**b**) TEM image of ZnO/SnO_2_ NR and magnified view of the boundary region between the ZnO core and the SnO_2_ shell. (**c**) The corresponding FFT image of the SnO_2_ shell region and (**d**–**g**) STEM image of ZnO/SnO_2_ NR and corresponding elemental maps of Zn, Sn, and O, respectively.

To confirm the morphology and chemical composition of the ZnO/SnO_2_ core–shell structure in greater detail, TEM measurements were carried out. Figure 3a shows an HR-TEM image of a single ZnO/SnO_2_ NR separated from the NR array. The clear contrast between the core and the shell regions reveals that the core–shell structure of the NR was successfully formed, as expected based on the SEM analysis. Figure 3b shows a magnified view of the boundary region between the ZnO core and the SnO_2_ shell. The lattice spacing of 0.26 nm from the core region corresponds to the lattice spacing of the (002) plane of the crystalline ZnO, which indicates that the ZnO NRs were grown along the c-axis direction. In contrast, the SnO_2_ shell region had no apparent lattice fringe and the corresponding fast Fourier transform (FFT) images (Figure 3c) indicate that the formed SnO_2_ shell had an amorphous structure. Figure 3d–g shows scanning transmission electron microscopy (STEM) images of the ZnO/SnO_2_ NR and the corresponding elemental maps of the Zn, Sn, and O, respectively. The Zn atoms were located in the core region and the Sn atoms covered the Zn core region. Based on the SEM and HR-TEM analysis, the crystal structure of the as-synthesized core–shell NRs was confirmed to be composed of crystalline ZnO/a-SnO_2_ NRs.

**Figure 4:**
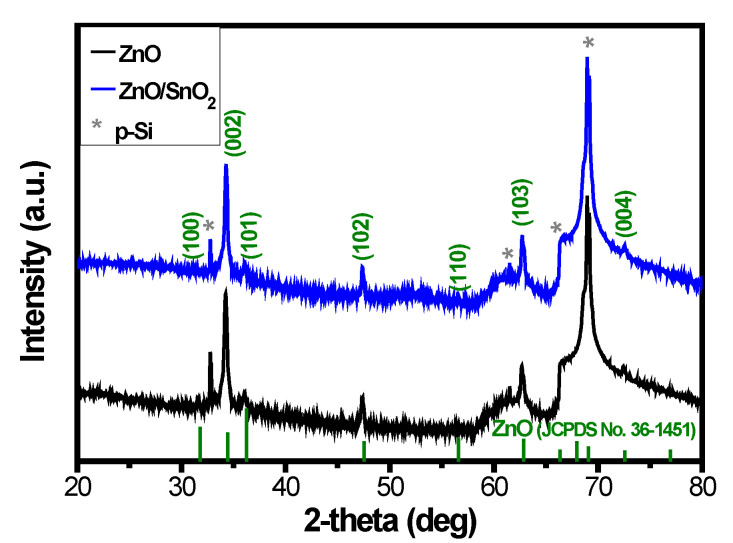
The XRD patterns of ZnO NRs and ZnO/SnO_2_ NRs.

To further investigate the crystal structure of the ZnO/a-SnO_2_ core–shell NRs, XRD analysis was performed. The XRD patterns of the ZnO NRs, as shown in Figure 4, correspond to the wurtzite structure of ZnO (JCPDS No.36-1451). After deposition of the SnO_2_ shell layer, no additional XRD peaks were assigned for the SnO_2_, suggesting that the SnO_2_ shell layer had an amorphous structure, which corresponds with the TEM analysis results.

The amorphous structure of the SnO_2_ shell layer which formed on the ZnO can be explained by the process temperature. As expected (based on the TEM and XRD measurements), using a low synthesis temperature below the thermal energy required to form a crystallized structure can lead to the amorphous phase formation of the grown SnO_2_. During the formation of the shell layer at a sufficiently low temperature, there is a lack of driving forces for the surface diffusion of foreign atoms, and even foreign atoms can stick close to the position where they first arrive. This leads to the formation of an amorphous phase. Therefore, the lack of driving forces for the surface diffusion of tin and oxygen ions can induce the amorphous growth of SnO_2_. 

**Figure 5:**
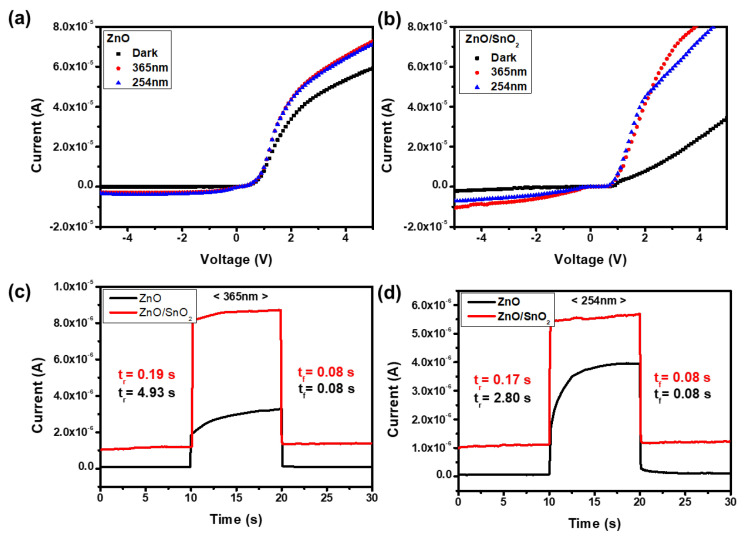
The I–V characteristics of (**a**) ZnO and (**b**) ZnO/SnO_2_ PDs under dark, 365 nm, and 254 nm UV light conditions. Time-domain photoresponse of ZnO and ZnO/SnO_2_ PDs under (**c**) 365 nm UV light and (**d**) 254 nm UV light at a fixed bias condition of −5 V.

To evaluate the performance of ZnO/a-SnO_2_ core–shell NRs UV PDs, I–V characteristics and time-domain photoresponse values were measured. Figure 5a–c shows the I–V characteristics of the ZnO/a-SnO_2_ PD (red line) and the bare ZnO PD (black line) under dark, 365 nm UV light, and 254 nm UV light conditions, respectively. There are several key figures of merit (FOMs) that can be used to analyze the performance of PDs, such as the photocurrent and responsivity. The photocurrent is defined as ∆I = I_light_ − I_dark_, where I_light_ is the current under light illumination and I_dark_ is the current under dark conditions. The photocurrent values of the ZnO/a-SnO_2_ NRs PD were 8.57 × 10^−6^ A and 4.86 × 10^−6^ A under 365 nm UV light and 254 nm UV light illumination at −5 V, respectively, whereas those of the bare ZnO NRs PD were 2.75 × 10^−6^ A and 3.11 × 10^−6^ A. Another key FOM for PDs, i.e., responsivity R, is expressed using the following equation: R = ∆I/PS. Here, P is the incident light intensity and S is the effective illuminated area. The responsivities of the fabricated devices were calculated based on the measured photocurrents and the effective illuminated area, 1.5 × 1.5 cm^2^. Under 365 nm UV light illumination, the responsivity of the ZnO/a-SnO_2_ PD was 6.24 × 10^−3^ A/W at −5 V, whereas the ZnO PD had a value of 2 × 10^−3^ A/W at −5 V. Under 254 nm UV light, the responsivity of the ZnO/a-SnO_2_ PD was 5.4 × 10^−3^ A/W at −5 V, whereas the ZnO PD showed a value of 3.46 × 10^−3^ A/W at −5 V. The responsivity was improved by 3.1 and 1.6 times under 365 nm and 254 nm UV light illumination, respectively, by using the a-SnO_2_ shell layer.

To investigate the enhancement of the photoresponse speed when the a-SnO_2_ shell layer was applied to the ZnO NRs PDs, the time-domain photoresponses were measured at a fixed bias condition of −5 V, as shown in Figure 5c,d. The response speed for incident light can be expressed by the rise time, T_r_ (or fall time, T_f_), which is defined as the time required for the photocurrent to increase from 10% to 90% of its maximum current (or decrease from 90% to 10%). Under 365 nm UV light, the ZnO/a-SnO_2_ PD exhibited a faster rise time, i.e., 0.19 s, compared to that of the ZnO PD (4.93 s), while the fall time remained stable (about 0.08 s), as shown in Figure S1a. Under 254 nm UV light, the ZnO/a-SnO_2_ PD also exhibited a faster rise time, i.e., 0.17 s, compared to that of bare ZnO PD (2.80 s), while the fall time again remained substantially stable at about 0.08 s, as shown in Figure S1b. To compare the performances of our a-SnO/ZnO NRs-based device with previously reported ZnO- and SnO_2_-based PDs, we summarized the key FOMs in Table S1. As demonstrated, the ZnO/a-SnO_2_ PD showed a dramatically improved performance as a UV PD in comparison with the ZnO PD. The rise and fall times, which are critical shortcomings of ZnO PDs, were considerably improved by 26 times and 16 times, respectively.

**Figure 6:**
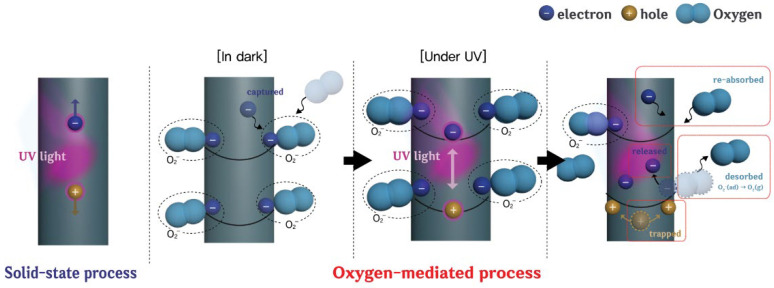
The solid-state process and oxygen-mediated mechanism of the ZnO-based PD.

The significant improvement in the photoresponse speed by using the a-SnO_2_ shell layer for ZnO/a-SnO_2_ UV PDs can be explained by the surface passivation effect. The photodetection process occurring on the PD can be divided into two parts, i.e., a bulk-related process and a surface-related process [23], as shown in Figure 6. The photodetection process in the bulk can be referred to as a solid-state process, meaning that the electron–hole pairs (EHPs) generated by the adsorption of light on the surface of the PD directly gives rise to the photocurrent by means of a built-in or externally applied potential. However, the situation that occurs on the surface is different from the situation in the bulk. Due to the high surface–volume ratio of NRs, interactions with environmental species, such as oxygen, can significantly impact the device performances of PDs. The main photodetection process present on the ZnO surface can be referred to as an oxygen-mediated process [7]. In dark conditions, oxygen is absorbed on the ZnO surface and simultaneously captures electrons from the conduction band of the ZnO [O_2_ + e → O_2_^−^]. As a result, a depletion region is formed and band-bending occurs. In the UV light illumination state, EHPs are generated and photogenerated holes are drawn to the surface of the ZnO due to the band bending. Then, negatively charged oxygen is desorbed by the photogenerated holes, releasing captured electrons that participate in electrical conduction [h^+^ + O_2_^−^ → O_2_]. This oxygen-mediated process has characteristics that adversely affect the performance of PDs. It is also a relatively slow process compared to the solid-state process where photogenerated carriers directly participate in the photocurrent without being captured or released by oxygen for photodetection [11,14]. Accordingly, ZnO-based UV PDs suffer from slow photoresponse speeds. However, after passivation of the ZnO surface by the a-SnO_2_ shell layer, the photoresponse speed of the device can be improved by reducing the contribution of the slow oxygen-mediated process. In addition, there are many subgap states formed by defect sites, which deteriorate the performances of PDs. However, by forming the a-SnO_2_ shell layer, those can be reduced and the light responsivity of the PD can be improved.

**Figure 7:**
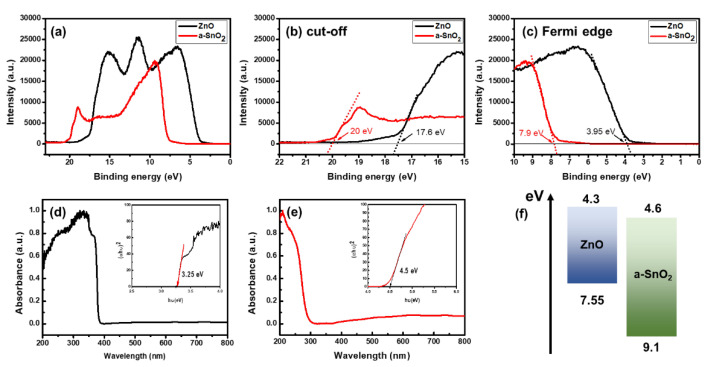
(**a**) UPS spectra of ZnO and SnO_2_, (**b**) the cut-off energy region, and (**c**) the Fermi edge region of ZnO and SnO_2._ UV-Vis measurements of (**d**) ZnO NRs and (**e**) SnO_2_ NRs. The insets of (**d**) and (**e**) show the bandgaps of ZnO NRs and SnO_2_ NRs. (**f**) Energy band diagram of ZnO NRs and SnO_2_ NRs.

To investigate the reason for the improvement in the light responsivity and the role of the a-SnO_2_ shell layer in the ZnO/a-SnO_2_ core–shell UV PD, we measured the ultraviolet photoelectron spectroscopy (UPS), as shown in Figure 7a–c. The Fermi level (E_F_) and valence band maximum (E_VB_) is calculated using the following equation: E_F_ = E_cut-off_ − 21.2 eV, E_VB_ = E_F_ − E_F,edge_ (Fermi edge). The optical band gap (E_g_) can be obtained from the UV–Vis spectroscopy results shown in Figure 7d,e. Based on the UPS and UV–Vis spectroscopy data, the calculated energy levels of the E_VB_ and E_g_ are 7.55 eV and 3.25 eV in the ZnO, and 9.1 eV and 4.5 eV in the a-SnO_2_. The band diagram of the ZnO/a-SnO_2_ based on the observed energy levels is shown in Figure 7f, which forms a type II heterojunction. When the EHPs are generated by incident photons, generated electrons prefer to move from the ZnO to SnO_2_ and the holes prefer to move from the SnO_2_ to ZnO. The type II heterojunction band structure effectively separates photogenerated electrons and holes, thus suppressing recombination [32]. Therefore, the prolonged lifetime of electrons and holes can contribute to the enhanced light responsivity of the ZnO/a-SnO_2_ UV PD. In addition, the a-SnO_2_ shell layer, which has a higher optical band gap (~4.5 eV) than the ZnO (~3.25 eV), can act as a window layer for the ZnO core without interfering with the absorption of the ZnO core material.

**Figure 8:**
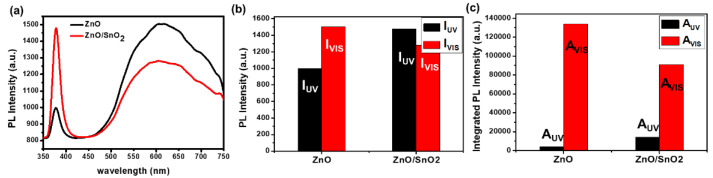
(**a**) Photoluminescence spectra of ZnO NRs, and ZnO/SnO_2_ NRs. (**b**) The intensity ratio of the UV and visible luminescence of ZnO and ZnO/SnO_2_ PDs and (**c**) the integrated intensity ratio of the UV and visible luminescence of ZnO and ZnO/SnO_2_ PDs.

Finally, to investigate whether the surface states of the ZnO were effectively passivated by the deposition of the SnO_2_ shell layer, photoluminescence (PL) measurements were carried out at room temperature with a 325 nm He-Cd lighting source. Generally, the broad, deep-level luminescence of ZnO around 550 to 700 nm in the visible region originates from surface defect states [33]. Therefore, if the surface of the ZnO is successfully passivated, then the visible luminescence of the ZnO should be reduced. Figure 8 shows the PL spectra obtained from the bare ZnO and ZnO/a-SnO_2_ core–shell NRs. After the deposition of the a-SnO_2_ on the surface of the ZnO NRs, the band edge emission of the ZnO/a-SnO_2_ NRs at 377 nm significantly increased and the visible luminescence of the ZnO/a-SnO_2_ was considerably decreased. When we compared the ratio of I_UV_/I_VIS_, which indicates the PL intensity ratio of UV and visible luminescence centered at 377 nm and 610 nm, the I_UV_/I_VIS_ value of the ZnO/a-SnO_2_ NRs was increased by a factor of 1.74 compared to that of the ZnO NRs (0.66 → 1.15). In addition, the value of A_UV_/A_VIS_, which refers to the integrated PL intensity ratio of UV and visible luminescence, was increased by a factor of 5.33 for the ZnO/a-SnO_2_ NR compared to that of the ZnO NRs (0.03 → 0.16). Moreover, as is expected from the UV–Vis measurements at Figure 7e, the a-SnO_2_ shows no apparent absorption in the range of 350 to 750 nm, indicating that the a-SnO_2_ shell layer can effectively act as a window layer for the ZnO core. Although the exact origin of the visible luminescence of the ZnO remains controversial, it is reasonable to assume that surface states may play a role. Several researchers have reported that the visible luminescence of ZnO can be reduced by surface passivation [25,34]. Based on the PL results, we can conclude that the ZnO surface was successfully passivated by the a-SnO_2_, which may be the main mechanism for the enhancement of the light response speed and responsivity characteristics of ZnO UV PDs. 

## 4. Conclusions

In summary, we fabricated ZnO/a-SnO_2_ core–shell UV PDs to improve the photoresponse speed of bare ZnO PDs without deteriorating the light responsivity. The rise time of the ZnO/a-SnO_2_ PD was improved by a factor of 26 compared to the ZnO PD under 365 nm UV light irradiation. Simultaneously, the light responsivity of the ZnO/a-SnO_2_ PD compared to that of the ZnO PD increased by 3.1 times under 365 nm UV light. Surface passivation of the ZnO core by the formation of an a-SnO_2_ shell layer can suppress the slow oxygen-mediated process, thereby enhancing the response speed under UV light. PL measurements (I_UV_/I_VIS_) clearly indicated that the surface states of the ZnO were significantly reduced by the a-SnO_2_ passivation. Due to the enhanced light response speed and light responsivity in UV PDs made with a relatively low fabrication temperature, the ZnO/a-SnO_2_ core–shell structure is applicable to various ZnO-based PDs that require a fast light response speed, high responsivity, and low fabrication temperature.

## Data Availability

Not applicable.

## Supplementary Materials

The following are available online at https://www.mdpi.com/article/10.3390/s21186124/s1, Figure S1: Magnified time-domain photoresponse of ZnO and ZnO/SnO_2_ PDs under (a) 365 nm and (b) 254 nm UV light at a fixed bias condition of −5 V, Table S1: Summary table of other ZnO- or SnO_2_-based photodetectors previously reported.

## References

[1] Monroy E., Omnès F., Calle F. (2003). Wide-bandgap semiconductor ultraviolet photodetectors. Semicond. Sci. Technol..

[2] Omnès F., Monroy E., Muñoz E., Reverchon J.-L. (2007). In Wide bandgap UV photodetectors: A short review of devices and applications. Proc. SPIE.

[3] Sang L., Liao M., Sumiya M. (2013). A comprehensive review of semiconductor ultraviolet photodetectors: From thin film to one-dimensional nanostructures. Sensors.

[4] Liang S., Sheng H., Liu Y., Huo Z., Lu Y., Shen H. (2001). ZnO Schottky ultraviolet photodetectors. J. Cryst. Growth.

[5] Yang P., Yan H., Mao S., Russo R., Johnson J., Saykally R., Morris N., Pham J., He R., Choi H.J. (2002). Controlled growth of ZnO nanowires and their optical properties. Adv. Funct. Mater..

[6] Yang T., Park S.-J., Kim T.G., Shin D.S., Park J. (2017). Ultraviolet photodetector using pn junction formed by transferrable hollow n-TiO_2_ nano-spheres monolayer. Opt. Express.

[7] Hu L., Yan J., Liao M., Wu L., Fang X. (2011). Ultrahigh external quantum efficiency from thin SnO_2_ nanowire ultraviolet photodetectors. Small.

[8] Wu J.-M., Kuo C.-H. (2009). Ultraviolet photodetectors made from SnO_2_ nanowires. Thin Solid Film..

[9] Zhai T.Y., Li L., Wang X., Fang X.S., Bando Y., Golberg D. (2010). Recent developments in one-dimensional inorganic nanostructures for photodetectors. Adv. Funct. Mater..

[10] Shen G., Chen D. (2010). One-dimensional nanostructures for photodetectors. Recent Pat. Nanotechnol..

[11] Lien D.H., Retamal J.R.D., Ke J.J., Kang C.F., He J.H. (2015). Surface effects in metal oxide-based nanodevices. Nanoscale.

[12] Wang Y., Wang P., Zhu Y.K., Gao J.R., Gong F., Li Q., Xie R.Z., Wu F., Wang D., Yang J.H. (2019). High performance charge-transfer induced homojunction photodetector based on ultrathin ZnO nanosheet. Appl. Phys. Lett..

[13] Zhang Z.M., Ning Y., Fang X.S. (2019). From nanofibers to ordered ZnO/NiO heterojunction arrays for self-powered and transparent UV photodetectors. J. Mater. Chem. C.

[14] Liu S., Li M.Y., Zhang J., Su D., Huang Z., Kunwar S., Lee J. (2020). Self-assembled Al nanostructure/ZnO quantum dot heterostructures for high responsivity and fast UV photodetector. Nanomicro Lett..

[15] You D., Xu C., Zhang W., Zhao J., Qin F., Shi Z. (2019). Photovoltaic-pyroelectric effect coupled broadband photodetector in self-powered ZnO/ZnTe core/shell nanorod arrays. Nano Energy.

[16] Zhang L., Wang Y., Wu H., Hou M., Wang J., Zhang L., Liao C., Liu S., Wang Y. (2019). A ZnO nanowire-based microfiber coupler for all-optical photodetection applications. Nanoscale.

[17] Zheng M., Gui P., Wang X., Zhang G., Wan J., Zhang H., Fang G., Wu H., Lin Q., Liu C. (2019). ZnO ultraviolet photodetectors with an extremely high detectivity and short response time. Appl. Surf. Sci..

[18] You D., Xu C., Zhao J., Qin F., Zhang W., Wang R., Shi Z., Cui Q. (2019). Single-crystal ZnO/AlN core/shell nanowires for ultraviolet emission and dual-color ultraviolet photodetection. Adv. Opt. Mater..

[19] Zhou J., Gu Y., Hu Y., Mai W., Yeh P.-H., Bao G., Sood A.K., Polla D.L., Wang Z.L. (2009). Gigantic enhancement in response and reset time of ZnO UV nanosensor by utilizing Schottky contact and surface functionalization. Appl. Phys. Lett..

[20] Soci C., Zhang A., Xiang B., Dayeh S.A., Aplin D.P.R., Park J., Bao X.Y., Lo Y.H., Wang D. (2007). ZnO nanowire UV photodetectors with high internal gain. Nano Lett..

[21] Liu Y., Gorla C., Liang S., Emanetoglu N., Lu Y., Shen H., Wraback M. (2000). Ultraviolet detectors based on epitaxial ZnO films grown by MOCVD. J. Electron. Mater..

[22] Park J., Shin D.S., Kim D.H. (2014). Enhancement of light extraction in GaN-based light-emitting diodes by Al_2_O_3_-coated ZnO nanorod arrays. J. Alloy. Compd..

[23] Park J., Ryu H., Son T., Yeon S. (2012). Epitaxial growth of ZnO/InN core/shell nanostructures for solar cell applications. Appl. Phys. Express.

[24] Tian W., Zhai T., Zhang C., Li S.L., Wang X., Liu F., Liu D., Cai X., Tsukagoshi K., Golberg D. (2013). Low-cost fully transparent ultraviolet photodetectors based on electrospun ZnO-SnO_2_ heterojunction nanofibers. Adv. Mater..

[25] Qin L.Q., Shing C., Sawyer S., Dutta P.S. (2011). Enhanced ultraviolet sensitivity of zinc oxide nanoparticle photoconductors by surface passivation. Opt. Mater..

[26] Cossuet T., Resende J., Rapenne L., Chaix-Pluchery O., Jiménez C., Appert E., Muñoz-Rojas D., Consonni V., Deschanvres J.-L. (2018). ZnO/CuCrO_2_ core–shell nanowire heterostructures for self-powered UV photodetectors with fast response. Adv. Funct. Mater..

[27] Zhou M., Wu B., Zhang X., Cao S., Ma P., Wang K., Fan Z., Su M. (2020). Preparation and UV photoelectric properties of aligned ZnO–TiO_2_ and TiO_2_–ZnO core–shell structured heterojunction nanotubes. ACS Appl. Mater. Interfaces.

[28] Xiong L., Guo Y., Wen J., Liu H., Yang G., Qin P., Fang G. (2018). Review on the application of SnO_2_ in perovskite solar cells. Adv. Funct. Mater..

[29] Wali Q., Fakharuddin A., Jose R. (2015). Tin oxide as a photoanode for dye-sensitised solar cells: Current progress and future challenges. J. Power Sources.

[30] Yoon H., Shin D.S., Babu B., Kim T.G., Song K.M., Park J. (2017). Control of copper nanowire network properties and application to transparent conducting layer in LED. Mater. Des..

[31] Kwon D.K., Lee S.J., Myoung J.M. (2016). High-performance flexible ZnO nanorod UV photodetectors with a network-structured Cu nanowire electrode. Nanoscale.

[32] Ouyang W., Teng F., He J.H., Fang X. (2019). Enhancing the photoelectric performance of photodetectors based on metal oxide semiconductors by charge-carrier engineering. Adv. Funct. Mater..

[33] Shalish I., Temkin H., Narayanamurti V. (2004). Size-dependent surface luminescence in ZnO nanowires. Phys. Rev. B.

[34] Guo L., Yang S.H., Yang C.L., Yu P., Wang J.N., Ge W.K., Wong G.K.L. (2000). Highly monodisperse polymer-capped ZnO nanoparticles: Preparation and optical properties. Appl. Phys. Lett..

