# Supplementary material for: Improved Photoresponse Characteristics of a ZnO-Based UV Photodetector by the Formation of an Amorphous SnO2 Shell Layer
Source: Sensors (Basel). 2021 Sep 13;21(18):6124. doi: 10.3390/s21186124 (PMC8473163; doi:10.3390/s21186124)
Supplement: Supplementary file 1 [file sensors-21-06124-s001.zip › sensors-1348323-supplementary.pdf]

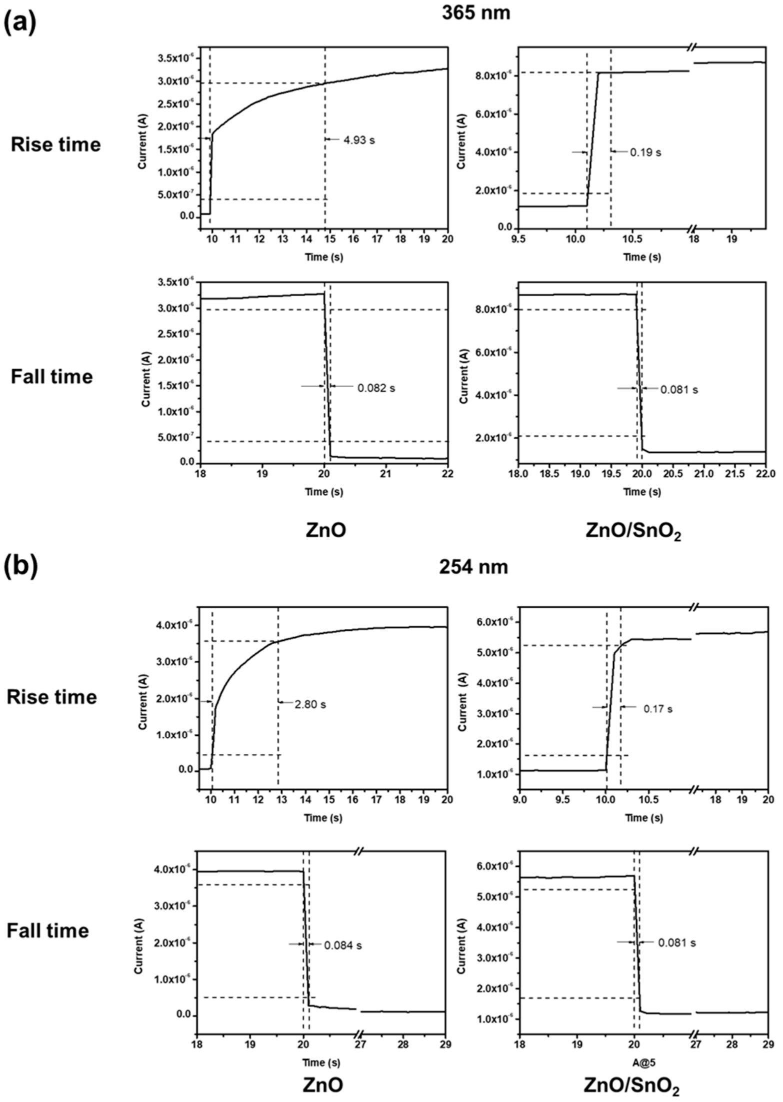

Figure S1. Magnified time-domain photoresponse of ZnO and ZnO/SnO_2 PDs under (a) 365nm and (b) 254nm UV light at a fixed bias condition of –5V.

Table S1. Summary table of other ZnO- or SnO_2- based photodetectors previously reported

Material	Wavelength	Bias	Light intensity	Dark current	Photocurrent	Iph/ Idark	Responsivity	Rise time	Fall time	Ref.
ZnO(NW) + SIG	365nm	5V	30μW/cm^2	0.15nA	0.68μA	4533	-	-	0.3s	1
ZnO(NR)	-	5V	-	-	-	2060	1.12x10^3 A/W	3s	1s	2

ZnO(NS)-HfO2	450nm	1V	-	1pA	-	-	6.83x10^3 A/W	0.3s	0.31s	3
ZnO(NF)-NiO	-	0V	-	-	-	-	0.415 mA/W	7.5s	4.8s	4
rGO+ZnO+GQD	-	2V	-	35.6μA/cm^2	128 μA/cm^2	3.59	12.8 A/W	4.26s	10.97s	5
ZnO(film)	-	-	-	-	-	>10^2	2.2 A/W	0.2s	0.3s	6
ZnO(NP film)	365nm	-2V	0.1 mW/cm2	0.66μA	80μA	120	-	64ms	70ms	7
ZnO-SnO2(single)	300nm	2V	-	200fA	300pA	1500		162.9s	75.5s	8
SnO2	365nm	0.4 mV	0.2mWcm^{-2}	32pA	1.43nA	44	15	250s	506s	9
SnO2(amorphous)	370nm	0V	107Wcm^{-2}	-	-	6018	0.36	0.72s	1.78s	10
Z-S nanofiber	300nm	10V	0.45	1.7pA	7.9nA	4600	-	32.2s	7.8s	11
S-Z Nanowires	365nm	5V	17100	35.3μA	53.5μA	1.52	-	>100s	328s	12
S-Z Brush-like	325nm	5V	24	3μA	40μA	13.3	-	>10s	>10s	13
ZnO – SnO2	365nm	-5V		2.07μA	10.64μA	5.14	5.4 mA/W	0.19s	0.08s	Our work
	254nm	-5V		2.07μA	6.93μA	3.35	3.46 mA/W	0.17s	0.08s	

*NF=Nanofiber , *NP=Nanoparticle, *NW=Nanowire, *NR=Nanorod, *NS=Nanosheet

Reference

1. Yang, F.; Zheng, M.; Zhao, L.; Guo, J.; Zhang, B.; Gu, G.; Cheng, G.; Du, Z. The high-speed ultraviolet photodetector of ZnO nanowire Schottky barrier based on the triboelectric-nanogenerator-powered surface-ionic-gate. Nano Energy, 2019, 60, 680-688.
2. Shan, C.; Zhao, M.; Jiang, D.; Li, Q.; Li, M.; Zhou, X.; Duan, Y.; Wang, N.; Deng, R. Improved responsivity performance of ZnO film ultraviolet photodetectors by vertical arrays ZnO nanowires with light trapping effect. *Nanotechnology, 2019, 30,* 305703.
3. Wang, Y.; Wang, P.; Zhu, Y.; Gao, J.; Gong, F.; Li, Q.; Xie, R.; Wu, F.; Wang, D.; Yang, J.; Fan, Z.; Wang, X.; Hu, W. High performance charge-transfer induced homojunction photodetector based on ultrathin ZnO nanosheet. Appl. Phys. Lett., 2019, 114,

011103.
4. Zhang, Z.; Ning, Y.; Fang, X. From nanofibers to ordered ZnO/NiO heterojunction arrays for self-powered and transparent UV photodetectors. J. Mater. Chem. C, 2019, 7, 223-229.
5. Ko, K. B.; Ryu, B. D.; Han, M.; Hong, C. H.; Dinh, D. A.; Cuong, T. V. Multidimensional graphene and ZnO-based heterostructure for flexible transparent ultraviolet photodetector. Appl. Surf. Sci., 2019, 481, 524-530.
6. Zhang, W.; Jiang, D.; Zhao, M.; Duan, Y.; Zhou, X.; Yang, X.; Shan, C.; Qin, J.; Gao, S.; Liang, Q.; Hou, J. Piezo-phototronic effect for enhanced sensitivity and response range of ZnO thin film flexible UV photodetectors. J. Appl. Phys., 2019, 125, 024502.
7. Ling, C.; Guo, T.; Shan, M.; Zhao, L.; Sui, H.; Ma, S.; Xue, Q.; Oxygen vacancies enhanced photoresponsive performance of ZnO nanoparticles thin film/Si heterojunctions for ultraviolet/infrared photodetector. J. Alloys Compd., 2019, 797, 1224-1231.
8. Lou, Z.; Yang, X.; Chen, H.; Liang, Z. Flexible ultraviolet photodetectors based on ZnO–SnO2 heterojunction nanowire arrays. J. Semicond., 2018, 39, 024002.
9. Marimuthu, G.; Saravanakumar, K.; Jeyadheepan, K.; Razad, P. M.; Jithin, M.; Sreelakshmi, V. R.; Mahalakshmi, K. Influence of twin boundaries on the photocurrent decay of nanobranch and dense-forest structured SnO2 UV photodetectors. Superlattice. Microst., 2019, 128, 181-198.
10. Chetri, P.; Dhar, J. C. Self-powered UV detection using SnO2 nanowire arrays with Au Schottky contact. Mat. Sci. Semicon. Proc., 2019, 100, 123-129.
11. Tian, W.; Zhai, T.; Zhang, C.; Li, S. L.; Wang, X.; Liu, F.; Liu, D.; Cai, X.; Tsukagoshi,; Golberg, D.; Bando, Y. Low-cost fully transparent ultraviolet photodetectors based on electrospun ZnO-SnO2 heterojunction nanofibers. Adv. Mater., 2013, 25, 4625-4630.
12. Pan, K. Y.; Lin, Y. H.; Lee, P. S.; Wu, J. M.; Shih, H. C. Synthesis of SnO2-ZnO core-shell nanowires and their optoelectronic properties. J. Nanomater. 2012, 2012, 279245.
13. Dai, J.; Xu, C.; Guo, J.; Xu, X.; Zhu, G.; Lin, Y. Brush-like SnO2/ZnO hierarchical nanostructure: Synthesis, characterization and application in UV photoresponse. AIP Adv., 2013, 3, 062108.
